# Recombinant *Bacillus caldovelox* Arginase Mutant (BCA-M) Induces Apoptosis, Autophagy, Cell Cycle Arrest and Growth Inhibition in Human Cervical Cancer Cells

**DOI:** 10.3390/ijms21207445

**Published:** 2020-10-09

**Authors:** Sai-Fung Chung, Chi-Fai Kim, Ho-Yin Chow, Hiu-Chi Chong, Suet-Ying Tam, Yun-Chung Leung, Wai-Hung Lo

**Affiliations:** Department of Applied Biology and Chemical Technology, Lo Ka Chung Research Centre for Natural Anti-Cancer Drug Development and State Key Laboratory of Chemical Biology and Drug Discovery, The Hong Kong Polytechnic University, Hung Hom, Kowloon, Hong Kong, China; 16900402r@connect.polyu.hk (S.-F.C.); stephen.kim@polyu.edu.hk (C.-F.K.); hoyin.chow@polyu.edu.hk (H.-Y.C.); steve.h.c.chong@gmail.com (H.-C.C.); sabrinasy.tam@connect.polyu.hk (S.-Y.T.)

**Keywords:** apoptosis, arginase, autophagy, cell cycle arrest, cervical cancer cell

## Abstract

With our recent success in developing a recombinant human arginase drug against broad-spectrum cancer cell lines, we have explored the potential of a recombinant *Bacillus caldovelox* arginase mutant (BCA-M) for human cervical cancer treatment. Our studies demonstrated that BCA-M significantly inhibited the growth of human cervical cancer cells in vitro regardless of argininosuccinate synthetase (ASS) and argininosuccinate lyase (ASL) expression. Drug susceptibilities correlate well with the expressions of major urea cycle genes and completeness of L-arginine regeneration pathways. With the expressions of ASS and ASL genes conferring resistance to L-arginine deiminase (ADI) which is undergoing Phase III clinical trial, BCA-M offers the advantage of a broader spectrum of susceptible cancer cells. Mechanistic studies showed that BCA-M inhibited the growth of human cervical cancer cells by inducing apoptosis and cell cycle arrest at S and/or G_2_/M phases. Our results also displayed that autophagy served as a protective mechanism, while the growth inhibitory effects of BCA-M could be enhanced synergistically by its combination to the autophagy inhibitor, chloroquine (CQ), on human cervical cancer cells.

## 1. Introduction

Human cervical cancer is a disease that arises from the malignant growth of cells originating from the uterine cervix. Its development is strongly associated with the infection of high-risk type Human Papillomavirus (HPV) while the formation of invasive cancer requires the further accumulation of mutations and epigenetic alterations in certain tumor suppressor genes and oncogenes [1]. Despite the recent development of the HPV vaccine, cervical cancer is still the fourth most common and lethal cancer for women around the world [2]. With our recent success in developing an arginase-based anticancer drug against liver, lung, skin and colorectal cancers [3,4,5,6,7,8,9,10], we would like to explore the potential of a novel recombinant *Bacillus caldovelox* arginase mutant (BCA-M) [10] in the treatment of human cervical cancers.

**Figure 1:**
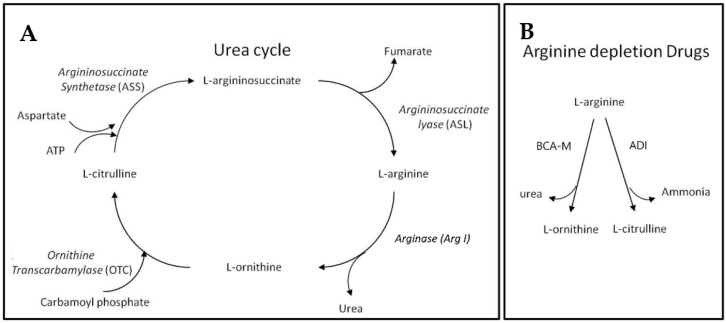
Urea cycle and L-arginine depletion drugs. (**A**) The urea cycle where L-arginine may be catabolized and regenerated. (**B**) The different actions of L-arginine depletion drugs resulting in different metabolites that may enter different steps of the urea cycle for L-arginine regeneration.

Before introducing BCA-M, we may first begin with the urea cycle (Figure 1). Apart from allowing animals to remove the relatively toxic ammonia while giving off urea, the urea cycle plays a key role in L-arginine metabolism. Within the urea cycle, arginase (Arg I) catalyzes the conversion of L-arginine into L-ornithine and urea while L-ornithine transcarbamylase (OTC) catalyzes the reaction of L-ornithine with carbamoyl phosphate to form L-citrulline [11]. From L-citrulline, L-arginine can be regenerated via the sequential catalytic actions of argininosuccinate synthetase (ASS) and argininosuccinate lyase (ASL), using up ATP while giving off fumarate during the processes [12,13]. The expressions of these urea cycle genes exhibit cell type specificity and the regeneration of L-arginine requires the cooperative metabolic functions of different body organs [12]. Apart from its role as a building block of peptides and proteins, L-arginine is involved in the synthesis of creatine, polyamine, proline, and cell-signaling molecules such as nitric oxide, L-glutamate, and agmatine [12,14]. L-arginine itself and the derived molecules are involved in a wide range of metabolic functions and cellular events. As a result of its biosynthesis, L-arginine is considered a conditional essential amino acid [12]. Many cancer cells are found to be auxotrophic for L-arginine and L-arginine depletion would leads to unbalanced growth, possibly because of their defective cell cycle regulations, resulting in cell death inevitably [15,16,17,18,19]. On the contrary, normal cells are resistant to L-arginine depletion, possibly because they may enter quiescence [16,17,20]. Our BCA-M is designed to continually catabolize L-arginine into L-ornithine and urea, thereby achieving and maintaining an L-arginine deprived condition that inhibits the growth of cancer cells, eventually leading to cell death. The difference in response to L-arginine deprivation between normal cells and malignant cells will not only improve the anticancer efficacy, but also benefit to the safety of arginase drugs. Theoretically, drug resistance may develop in cancer cells if they are capable of regenerating L-arginine via the urea cycle pathways. We hypothesize that the more complete the urea cycle pathways for regenerating L-arginine, the better the chance that the cells may utilize or scavenge the available resources to regenerate L-arginine, and thus the higher the resistance of the cancer cells towards L-arginine depletion will be. In this study, the susceptibility of human cervical cancer cells to BCA-M has been tested and compared to another L-arginine depletion drug—L-arginine deiminase (ADI, undergoing Phase III clinical trial [21])—which converts L-arginine into L-citrulline and ammonia. In the urea cycle, L-citrulline is one step closer than L-ornithine to being regenerated back into L-arginine, and therefore the cancer cells should have a better chance to be resistant to ADI.

For mechanistic studies, L-arginine depletion has been reported to induce cell cycle arrest and apoptosis in various susceptible cancer cells [4,5,16,22]. However, some authors reported that L-arginine-depletion-induced apoptosis may not be found in all cell types [16,17]. Therefore, we have attempted to study the effect of BCA-M on selected cervical cancer cell lines in these two areas. By comparing the induction of apoptosis and cell cycle arrest, we hope to further understand the complex cell death mechanism triggered by BCA-M treatment. On the other hand, autophagy is known to be an early cell protective mechanism upon nutrient deprivation [23,24,25], and therefore the inhibition of autophagy may improve the anticancer effect of BCA-M. In this regard, the lysosomotropic reagent chloroquine (CQ) is selected as a late-stage autophagy inhibitor that inhibits both autophagosome-lysosome fusion and lysosomal proteolysis [26]. In the present study, we attempted to confirm if autophagy also serves to protect cancer cells from BCA-M treatment and, more importantly, if the inhibition of autophagy, by CQ, may enhance the drug efficacy of BCA-M.

## 2. Results

### 2.1. Recombinant Bacillus Arginase Mutant (BCA-M) Suppressed the Growth of Human Cervical Cancer Cells

**Figure 2:**
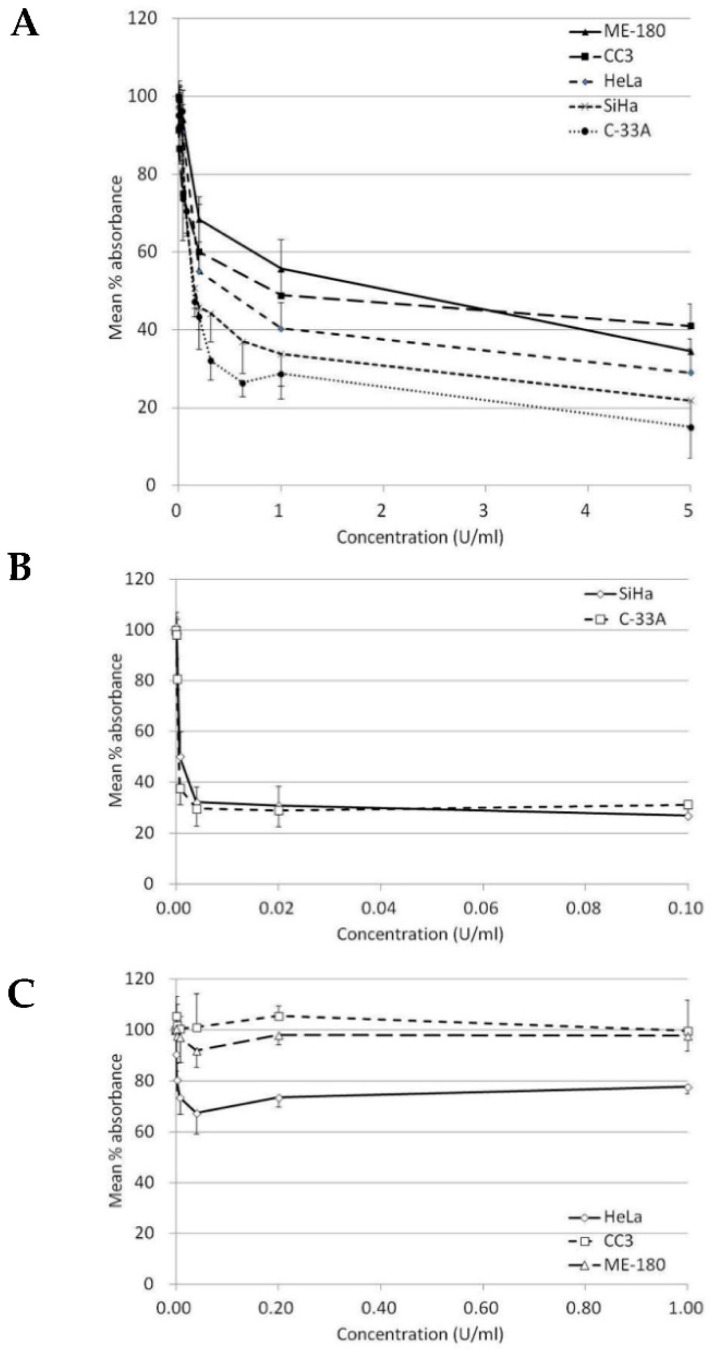
Growth inhibition of BCA-M and ADI on human cervical cancer cells. The results of cell proliferation assay are represented by the mean % absorbance and S.D. (**A**) BCA-M induced growth inhibition on human cervical cancer cells. (**B**) C-33A and SiHa cells were susceptible to ADI treatment. (**C**) HeLa, CC3 and ME-180 cells were resistant to ADI treatment even at a 10-fold higher maximum tested concentration, as in the susceptible cell lines.

Our results indicated that BCA-M significantly inhibited the growth of all five tested human cervical cancer cell lines after 72-h incubation (Table 1 and Figure 2A). The maximum percentage mean suppression (max. %) within the tested dose ranges for C-33A, SiHa, HeLa ME-180 and CC3 cells were 85%, 78%, 71%, 65% and 59%, respectively. IC_50_ values calculated from the non-linear regression model ranged from the lowest of 0.19 U/mL in C-33A cells to the highest of 1.42 U/mL in ME-180 cells. Both IC_50_ values and max. % indicated a similar trend of drug susceptibility in the order of C-33A > SiHa > HeLa > CC3 ~ ME-180. On the other hand, ADI only showed effective inhibition on the growth of SiHa and C-33A cells but were highly resisted by the remaining cell lines even at a 10-fold higher maximum concentration tested than the two sensitive cell lines (Table 1, Figure 2B,C).

### 2.2. Expression Profiles of Major Urea Cycle Genes in Human Cervical Cancer Cells

**Figure 3:**
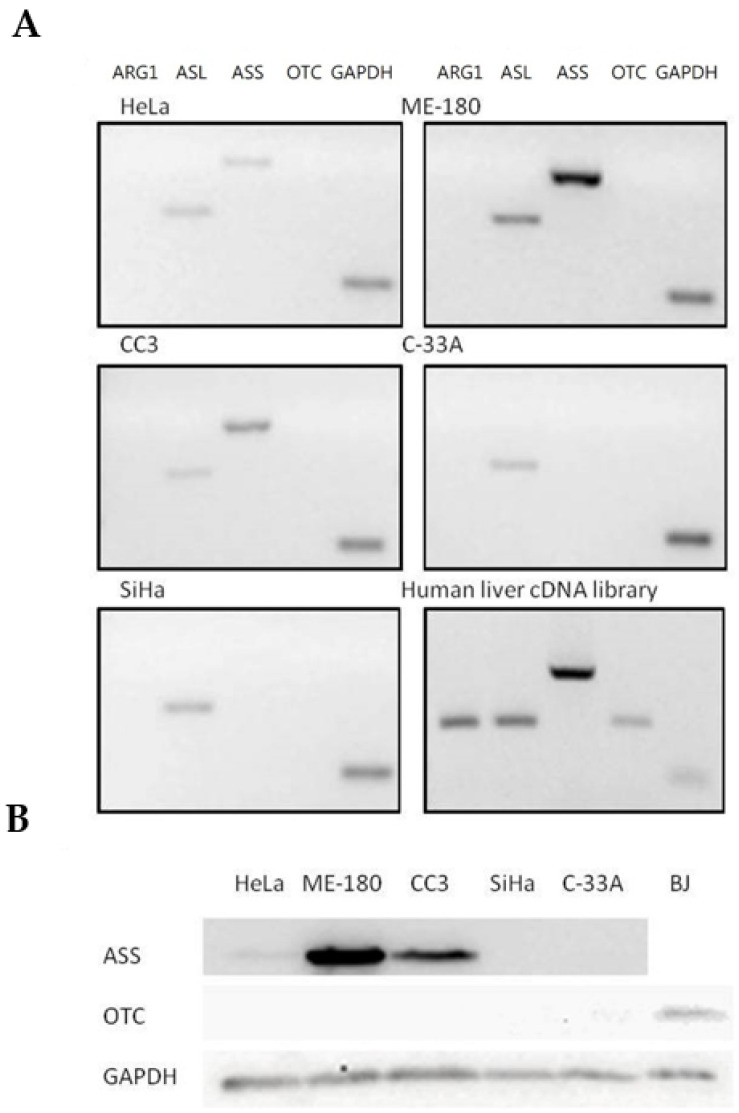
Expression profile of major urea cycle gene in human cervical cancer cells. (**A**) Transcriptional expression profiles in five human cervical cancer cell lines. The gene expression profiles of the cervical cancer cells and a positive control human liver cDNA library were determined using semi-quantitative RT-PCR and analyzed using agarose gel electrophoresis. (**B**) Protein expression of ASS and OTC was measured by Western blot analysis in five human cervical cancer cell lines. A positive control was performed using a normal human fibroblast cell line BJ. GAPDH served as internal control in all cell lines.

Expressions of major urea cycle genes were determined using semi-quantitative RT-PCR and normalized against the expression of GAPDH level (Table 1 and Figure 3A). ASL gene was expressed in all five cancer cell lines. Arg I and OTC expressions were undetectable in all five human cervical cancer cell lines, while the ASS gene was expressed only in three cell lines including ME-180, CC3 and HeLa. OTC expression, however, was detected using normal human liver cDNA library as a template, indicating that the undetectable OTC expression in the cervical cancer cells was due to deficient expression. Furthermore, the results were consistent with OTC protein expressions determined using Western blotting, where it was undetectable in all five cervical cancer cells but present in the BJ normal human fibroblast (Figure 3B). As for the ASS-positive cell lines, ME-180 and CC3 cells had a relatively stronger expression of ASS compared to the HeLa cells (Table 1). Again, the result was consistent with the ASS gene protein expression, where the ASS protein was detected with the corresponding levels in the three cell lines but undetectable in the others (Figure 3B). The relative expression level of ASL gene varied among different cell lines and was highest in the ME-180 cells, followed by SiHa and then the remaining cell lines (Table 1 and Figure 3A).

### 2.3. Over-Expression of OTC Conferred Significant Resistance Towards the Growth-Suppressive Effect of BCA-M on HeLa Cells

**Figure 4:**
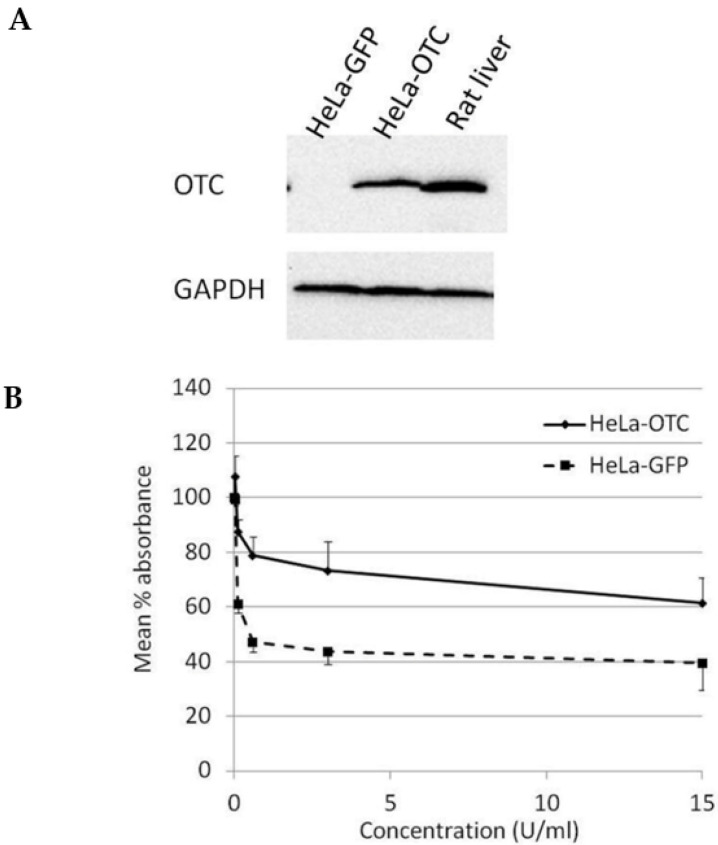
Over-expression of OTC conferred significant resistance towards the growth suppressive effect of BCA-M on HeLa cells. (**A**) The over-expression of OTC in transduced HeLa cells was confirmed by western blotting. Protein samples from HeLa cells expressing GFP and rat liver were used as negative and positive controls, respectively. (**B**) The results of cell proliferation assay for transduced HeLa cells expressing OTC or control GFP are represented by the mean % absorbance and S.D.

In order to study the effect of OTC expression on the susceptibility of cervical cancer cells towards BCA-M treatment, HeLa cells were transduced with OTC expression construct using recombinant adenovirus and confirmed with OTC expression using Western blotting (Figure 4A). The results from the cell proliferation assay showed that over-expression of OTC conferred significant resistance in HeLa cells towards the growth-suppressive effect of BCA-M in comparison to the GFP control and no IC_50_ value has been calculated (Figure 4B).

### 2.4. BCA-M-Induced Cell Cycle Arrest in Human Cervical Cancer Cells

**Figure 5:**
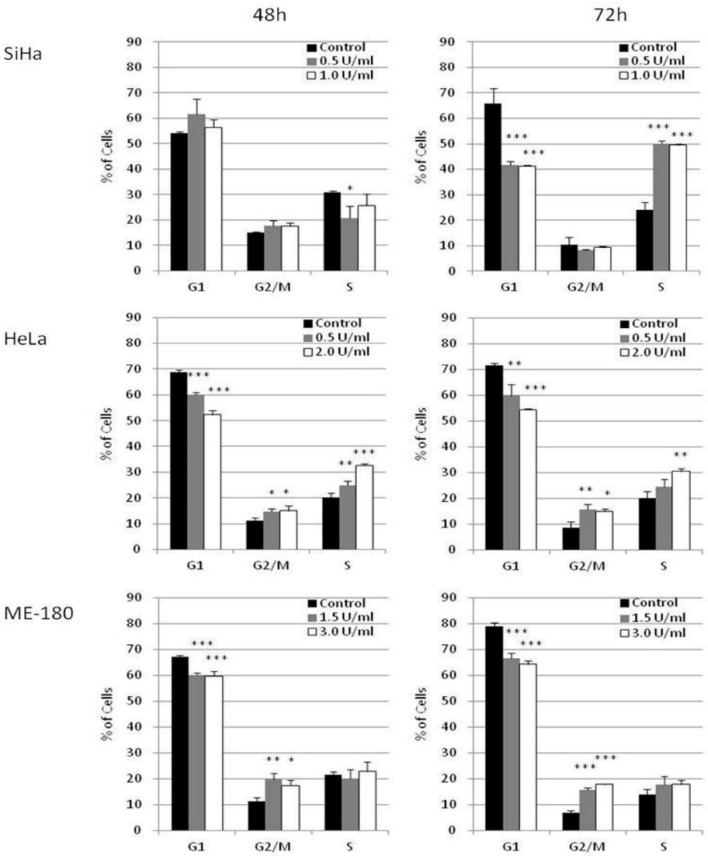
BCA-M induced cell cycle arrest in SiHa, HeLa and ME-180 cells. The effects of BCA-M on cell cycle phase distribution were determined using flow cytometric analysis with propidium iodide (PI) staining and RNase digestion. The results for percentage of cells in G_1_, G_2_/M and S phases were shown as means and S.D. One-way ANOVA indicated significant phase arrestment at S phase for SiHa cells, G_2_/M and S phases for HeLa cells and G_2_/M phase for ME-180 cells with *post hoc* Dunnett’s test showing p values for comparison between control and treatment as: * *p* < 0.05, ** *p* < 0.01, *** *p* < 0.001.

The effect of BCA-M on cell cycle phase distribution was studied in selected cervical cancer cells (SiHa, HeLa and ME-180) using flow cytometry with propidium iodide (PI) staining and RNase digestion (Figure 5 and Supplementary Materials Figures S1–S3). Owing to the different susceptibilities of these cancer cells, they were treated with different concentrations of BCA-M for 48 or 72 h in order to optimize the detection of cellular responses. Our results showed that BCA-M treatment induced a significant increase in the percentage of SiHa cells in the S phase sub-population after 72 h incubation, with a concomitant decrease in the percentage of cells at G_1_ phase but no significant change in that of the G_2_/M phase. For HeLa cells, BCA-M treatment resulted in a slight but significant increase in the percentage of cells in both G_2_/M and S phase sub-populations, suggesting that HeLa cells were arrested in these two phases. The increase in the percentage of S phase cells increased with the concentration of BCA-M after incubation for 48 and 72 h while the increase in the percentage of G_2_/M phase cells did not seem to be dose-dependent, or the effect has already reached a plateau. Interestingly for ME-180 cells, BCA-M treatment induced a significant increase in percentage, and thus probably arrestment, of the cells at G_2_/M phase only. Similar to that for HeLa cells, such an increase was slight and had yet to show a dose-dependent effect.

**Figure 6:**
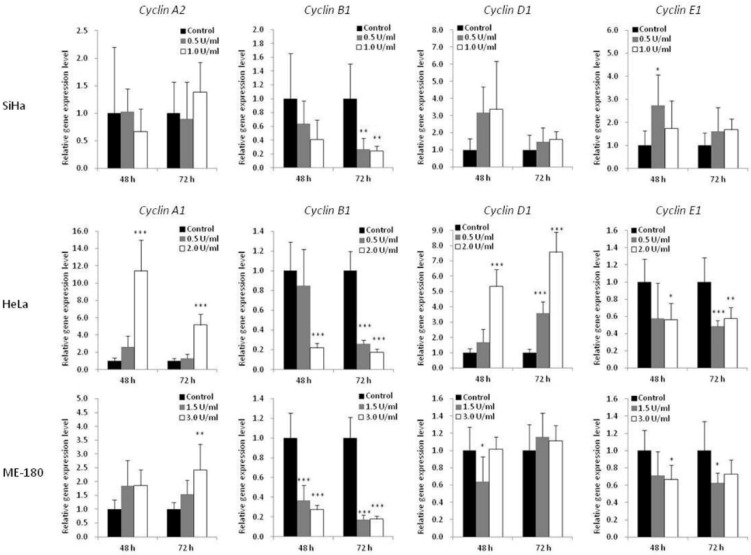
Effect of BCA-M on relative gene expression levels of various cyclins in SiHa, HeLa and ME-180 cells. The relative gene expression levels were determined using real-time PCR and normalized with the expression levels of the internal control (GAPDH, data not shown) and that of the respective genes in drug treatment control. The results were shown as means and S.D. One-way ANOVA with *post hoc* Dunnett’s test showing *p* values for comparison between control and treatment as: * *p* < 0.05, ** *p* < 0.01, *** *p* < 0.001.

To further understand the mechanism of BCA-M-induced cell cycle arrest, the transcriptional regulations of cyclin genes were analyzed using quantitative real-time PCR (Figure 6). The expression levels of cyclin A1 (CCNA1) were studied for HeLa and ME-180 cells while that of cyclin A2 (CCNA2) was studied for SiHa cells. The expression levels of cyclin B1 (CCNB1), cyclin D1 (CCND1) and cyclin E1 (CCNE1) were also determined for all these cell lines. For SiHa cells, BCA-M treatment resulted in the significant down-regulation of cyclin B1 expression after incubation for 72 h with both tested concentrations. For HeLa cells, 2.0 U/mL of BCA-M treatment significantly down-regulated the gene expressions of cyclin B1 and cyclin E1, with concomitant up-regulation of cyclin A1 and cyclin D1 after incubation for 48 and 72 h. For ME-180 cells, BCA-M significantly down-regulated cyclin B1 expression after treatment for 48 and 72 h with both tested concentrations. There was also a slight down-regulation of cyclin E1 and up-regulation of cyclin A1 expressions at both 48 and 72 h with or without statistical significance.

### 2.5. BCA-M Induces Apoptosis in Human Cervical Cancer Cells

**Figure 7:**
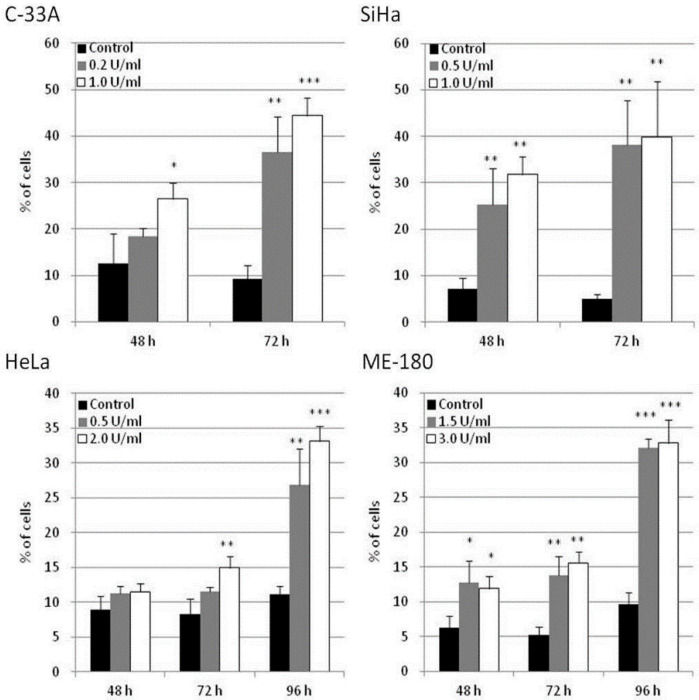
BCA-M-induced apoptosis in human cervical cancer cells. Apoptosis induction was detected using flow cytometric analysis with Annexin V-FITC and propidium iodide (PI) staining. The results for percentage of apoptotic cells were shown as means and S.D. One-way ANOVA with post hoc Dunnett’s test showing *p* values for comparison between control and treatment as: * *p* < 0.05, ** *p* < 0.01, *** *p* < 0.001.

The potential of apoptosis induction by BCA-M treatment in human cervical cancer cells was explored in C-33A, SiHa, HeLa and ME-180 cells using flow cytometric analysis with annexin V-FITC and propidium iodide staining. Owing to the different drug susceptibilities of these cancer cells and to optimize the detection of cellular responses, the cancer cells were treated with BCA-M at different doses and times chosen specifically for them. Our results showed that BCA-M significantly induced apoptosis in all the tested cell lines that have different drug susceptibilities and urea cycle gene expression profiles (Figure 7 and Figures S4–S7). For the relatively more susceptible C-33A and SiHa cells, BCA-M treatment resulted in a significant increase in the proportion of apoptotic cells in a time-dependent manner. For C-33A cells, massive apoptosis with 44.4 ± 3.8% of apoptotic cells was observed at 72 h incubation with 1.0 U/mL BCA-M treatment. For SiHa cells, massive apoptosis was also observed after 48 h incubation with 31.8 ± 3.8% of apoptotic cells and increased up to 39.9 ± 11.9% after 72 h with 1.0 U/mL BCA-M treatment. However, for the relatively less susceptible HeLa cells, a significant increase in the proportion of apoptotic cells was first observed after 72 h treatment with 2.0 U/mL BCA-M and massive apoptosis with a maximum of 33.2 ± 2.1% apoptotic cells appeared sharply after 96 h BCA-M treatment. Finally, for the relatively less susceptible ME-180 cells, although a significant increase in the proportion of apoptotic cells has been detected for all time points after 48 h, the percentage of apoptotic cells observed for 48 and 72 h was relatively low up to 15.6 ± 1.5%. Similar to HeLa cells, massive apoptosis was observed for ME-180 cells after 96 h with 3.0 U/mL BCA-M treatment resulting in a maximum of 32.8 ± 3.3% apoptotic cells. Therefore, the results for the extent of BCA-M-treatment-induced apoptosis showed certain correlations with the drug susceptibilities of the cell lines measured using MTT-based cell proliferation assays.

### 2.6. BCA-M-Induced Autophagy in HeLa and SiHa Cells and the Growth Inhibitory Effects Was Enhanced Synergistically by Its Combination to CQ

**Figure 8:**
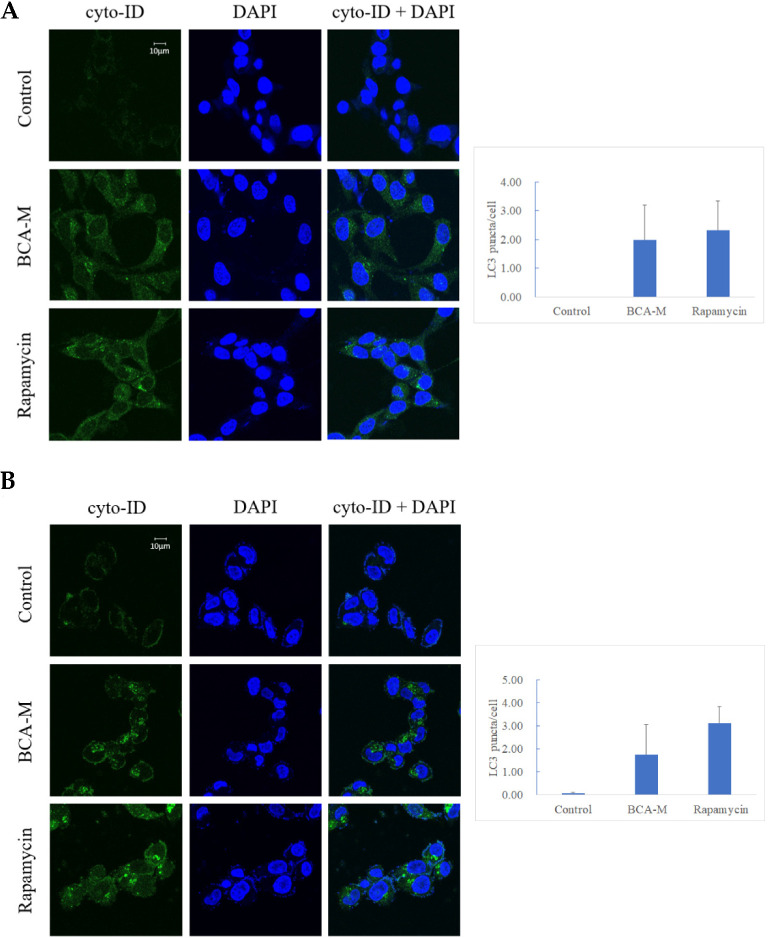
Fluorescent micrographs showing green punctate signals that represent autophagosome formation in (**A**) HeLa and (**B**) SiHa cells treated with BCA-M. Rapamycin was used as positive control. Quantification of the average number of LC3 puncta per cell was performed manually by counting obvious and large LC3 puncta (*n* = 3).

Our results showed that both HeLa and SiHa cells treated with 0.4–1.6 U/mL BCA-M for 1 h displayed early signs of autophagy (Figure S8). In order to enhance the effect of autophagy after the treatment of BCA-M, HeLa cells (Figure 8A) and SiHa cells (Figure 8B) treated with 4 U/mL BCA-M for 8 h resulted in a significant increase in the level of LC3-II puncta formation. The treatment of 500 nM rapamycin for 8 h (autophagy inducer) was used in the positive control during the experiment.

**Figure 9:**
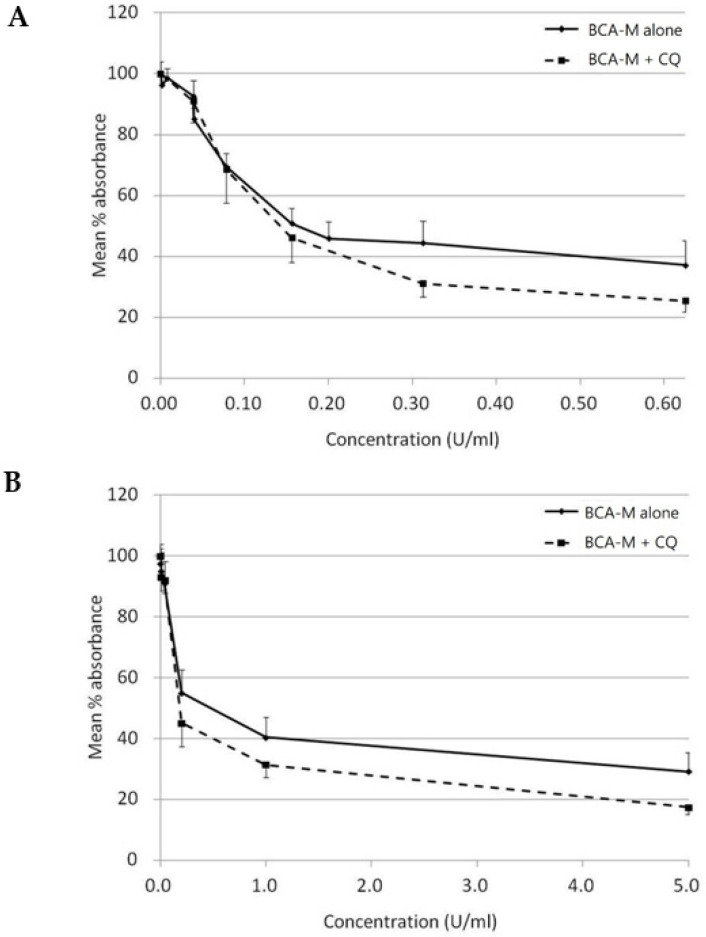
Growth inhibitory effect of BCA-M was enhanced synergistically by its combination to CQ on HeLa and SiHa human cervical cancer cells. The results of cell proliferation assay were represented by the mean % absorbance and S.D. (**A**) For SiHa cells, BCA-M was 2-fold serially diluted from 0.625 U/mL with or without combination to CQ starting at 6.25 μM and serially diluted along with BCA-M to maintain a constant ratio between the drugs. (**B**) For HeLa cells, constant drug ratio approach was also used with the starting concentration of BCA-M at 5 U/mL and CQ at 25 μM. In both cases, the combination of CQ enhanced the growth inhibitory effect of BCA-M.

On the other hand, with CQ inhibits autophagy, while autophagy is regarded as a protective mechanism against amino acid deprivation, we have attempted to evaluate the growth inhibitory effect of BCA-M alone or in combination to CQ using a constant drug-to-drug ratio approach on SiHa (Figure 9A) and HeLa (Figure 9B) cells. The growth inhibitory effect of CQ alone on both cell lines was also evaluated (Figure S9). Using the Chou-Talalay method [27,28], and calculated from the IC_50_ values, the combination indexes (C.I.) for BCA-M and CQ combinations were 0.78 ± 0.06 and 0.70 ± 0.21 for SiHa and HeLa cells, respectively (Table 2). The results indicated that the growth inhibitory effects of BCA-M were enhanced synergistically by its combination to CQ on both cell lines with negative and positive ASS expressions, respectively.

## 3. Discussion

In the present study, we have evaluated the drug susceptibilities of the human cervical cancer cells towards our BCA-M and ADI through maximum percentage mean suppression (max. %) and IC_50_ values. Our results clearly showed that drug susceptibility evaluated using both parameters correlates well with the expression profile of the major urea cycle genes (Table 1). All five tested human cervical cancer cell lines were highly susceptible to BCA-M. The C-33A and SiHa cells with undetectable mRNA and protein expressions of ASS and OTC genes were the most susceptible, with the lowest IC_50_ values and highest max. %. On the other hand, ME-180 and CC3 cells with relatively strong mRNA and protein expression levels of the ASS gene were the least susceptible with the highest IC_50_ values and lowest max. %. Although HeLa cells showed positive expressions of ASL and ASS genes, the relative expression level of ASS was much lower than that of ME-180 and CC3 cells for both mRNA and protein. The drug susceptibility of HeLa cells was in the middle, with around 70.9% of max. %. Although the expression level of ASL gene also varied among the cell lines, it did not seem to have a significant effect on the drug resistance while under the current expression levels. The results might be explained by the fact that ASS is the rate-limiting enzyme in the urea cycle and the L-citrulline-to-L-arginine regeneration pathways [29]. No one has ever reported on a situation of a complete absence of ASL enzyme activity [30]. In this respect, ASL seems to play a lesser role in the determination of the L-arginine deprivation susceptibility of cervical cancer cells.

On the other hand, cell lines (ME-180, CC3 and HeLa) with positive expression of ASL and ASS genes exhibit a contrasting difference in susceptibility to ADI when compared to cell lines (SiHa and C-33A) that do not express the ASS gene (Table 1, Figure 2B,C and Figure 3). The three former cell lines remain highly resistant to ADI even at a much higher dosage of ADI, equal to ten times the maximum tested concentration used on the SiHa and C-33A cell lines. For the ADI-resistant HeLa, CC3 and ME-180 cells, no IC_50_ value was calculable. The max. % within the tested dose range for these resistant cells was below 33% and seemed to reach the lowest plateau (Table 1). In fact, ASS expression has been found to correlate with resistance to ADI treatment in many cancer cell lines [24,31,32,33]. Furthermore, L-arginine deprivation might induce the expression of ASS in various cells types including certain melanoma, lymphoblastoma and epithelial cells [32,33]. By turning on the ASS expression, the cancer cells may develop resistance to ADI [32,33]. Although it is possible that turning on ASS expression may also reduce the drug efficacy of BCA-M, we have clearly shown that ASS-positive cell lines were still highly susceptible to BCA-M treatment, at least when they do not express OTC at the same time. Finally, ammonia generated from the ADI reaction is relatively more toxic than the reaction products of arginase [17]. When applied systemically, ADI may pose higher toxicity towards the healthy cells of the body. On the other hand, our results provided strong evidence that BCA-M is a promising broad spectrum arginine depletion-based anticancer drug candidate. In fact, we have previously demonstrated that a PEGylated form of BCA-M, with equivalent arginine depletion capability but more stable in vivo, had a remarkable anti-tumor effect on A549 lung tumor-bearing nude mice [10]. Our unpublished data also showed that repeated administrations of PEGylated BCA-M could maintain systemic arginine depletion for the whole testing period of a least a month in normal mice. With the in vitro, cancer-cell-suppressive IC_50_ values of BCA-M on the tested cervical cancer cells being lower than that of the A549 lung cancer cells [10], we expect the PEGylated BCA-M to be a feasible and promising anti-cervical cancer drug candidate. When applied systemically, PEGylated BCA-M would certainly have the overwhelming advantage of in vivo stability over BCA-M and should allow the PEGylated drug to be effective even for cancer at the metastatic stage. However, it would be interesting to further investigate if the accurate localized delivery and distribution maintenance of PEGylated BCA-M at high dosages to tumor tissues would enhance the suppressive efficacy of PEGylated BCA-M on relatively resistant tumors while minimizing any potential side effects on the host body.

In order to study the effect of BCA-M on cell cycle regulation of cervical cancer cells, we have employed flow cytometric cell cycle analysis with PI/RNase staining. The results showed that BCA-M arrested HeLa cells at both G_2_/M and S phase while it arrested SiHa cells at the S phase alone and arrested ME-180 cells at G_2_/M phase only, with statistical significance in each case (Figure 5). These three cervical cancer cell lines are known to contain high-risk serotype human papilloma virus (HPV) sequences in their DNA and expressed the E6 and E7 oncoproteins [34,35] It is very likely that the HPV-originated E6 and E7 resulted in defective cell cycle regulations that rendered the cells unable to be arrested at G_0_/_1_ phase via inactivation of the retinoblastoma protein (Rb) and p53 [35,36]. Further investigation into the mechanism of BCA-M-induced cell cycle arrest has been performed using quantitative real-time PCR to study the changes in the expression profile of selected cyclin genes (Figure 6). Cyclins are important co-enzymes required for the activation of cyclin-dependent kinases (CDK), and together they regulate cell cycle progression. Generally, in a typical normal eukaryotic cell undergoing a somatic division cycle, the levels of CDK proteins remain stable and it is the oscillation of the levels of cyclins that controls the activation of CDKs and plays an essential role in the regulation of cell cycle progression [37]. Among them, cyclin D plays a prominent role in G_1_ phase progression but, unlike other cyclins, it is expressed for as long as mitogenic stimulation persists [38]. After passing via the restriction point in late G_1_ phase, the cell is committed to progress through the cell cycle [39]. Cyclin E is expressed around the G_1_-S transition period, starting from the mid-G_1_ phase, peak at late G_1_ phase or the G_1_-S transition and then degraded sharply upon entering the S phase [40]. It is essential for the G_1_-S transition and serves a unique role in the initiation of DNA replication [38]. Cyclin A expression is initiated during the G_1_-S transition somewhat later than the initiation of cyclin E expression and its expression increases thereafter, reaching a peak in the S phase and persisting at a high level until its degradation just after the mitotic entry of the cell into prophase [40,41,42]. Cyclin A is important for S and G_2_ phase progression via interactions with different CDKs [43]. Cyclin B expression starts at low level in early G_2_/M phase and reaches its peak at G_2_/M transition. Then, it will be degraded in mitosis at the metaphase–anaphase transition [40]. For human cervical cancer, it has been reported that HPV infection is associated with promoter methylation of cyclin A1 (CCNA1) gene, thereby affecting its expression [44]. Kitkumthorn et al. [44] has reported that the expression level of cyclin A1 gene was low in SiHa cells, while it might be high in HeLa cells due to a demethylation process. Therefore, we have studied the expression of cyclin A2 for SiHa cells, and cyclin A1 for HeLa cells and ME-180 cells. For all these cell lines, the expression levels of cyclin B1, cyclin D1 and cyclin E1 were determined. For HeLa cells, the up-regulation of cyclin A1 gene and down-regulation of cyclin B1 gene expression point to the increase in the proportion of cells at S and/or early G_2_/M phases (Figure 6). The down-regulation of cyclin E1 gene expression suggested that the arrested cells were arrested at mid–late S and early G_2_/M phases. These results were consistent with flow cytometric analyses that showed an increase in the percentage of HeLa cells in both phases. For ME-180 and SiHa cells, BCA-M treatment induced a significant down-regulation of cyclin B1 gene expression with minor changes in the levels of other cyclin genes (Figure 6). The results suggested that these cells might be arrested at S and/or early G_2_/M phases. Interestingly, flow cytometric analyses showed that ME-180 cells were arrested at G_2_/M phase while SiHa cells have been arrested at S phase (Figure 5). Whether these differences were the results of the different drug mechanisms exhibited on these cancer cell lines or are simply due to the differences in responses of them remains to be clarified. Nevertheless, we demonstrated that BCA-M treatment in all tested human cervical cancer cell lines commonly induced a down-regulation of cyclin B1 gene expression and induced cell cycle arrest at S and/or G_2_/M phases. The results were strikingly similar to that of the recombinant human arginase treatment on human liver cancer cell lines where Hep3B cells were arrested at G_2_/M phase and HepG2 was arrested at S phase [4]. In fact, the L-arginine deprivation achieved using culture medium deficient in L-arginine has also been reported to lengthen the S and G_2_/M phases of many tumor cell lines, including the HeLa cells [16].

As in our present study, the effect of BCA-M treatment is consistent with that of L-arginine-deficient medium and the recombinant human arginase in bringing upon S and/or G_2_/M phase arrestment. However, when we compare the results for SiHa with HeLa or ME-180 cells, it is interesting to note that BCA-M treatment induced significant cell cycle arrestment at S-phase in the relatively more susceptible SiHa cells after treatment for 72 h, but not 48 h. In contrast, cell cycle arrestment for the less susceptible HeLa and ME-180 cells was observed after 48 h and continued along to 72 h. The results strongly suggested that, despite being less susceptible, HeLa and ME-180 cells showed a quicker response for cell cycle arrestment than the SiHa cells. When we take the results of apoptosis induction into account, BCA-M treatment induced massive apoptosis in SiHa cells after 48 h, while extensive apoptosis in HeLa and ME-180 cells were first observed at 96 h. Therefore, the results indicated that SiHa cells responded by apoptosis in a faster way than cell cycle arrestment while HeLa and ME-180 cells responded faster with cell cycle arrestment than apoptosis. Therefore, BCA-M showed multiple suppression mechanisms towards malignant cells. The results also strongly suggested that BCA-M-induced apoptosis may be delayed by the early response of cell cycle arrest, which is associated with the cell-dependent drug susceptibility. The drug susceptibility is, in turn, correlated with the expression profile of major genes and the availability of the corresponding L-arginine regeneration steps within the urea cycle.

Autophagy is a well-regulated catabolic process that involves the lysosomal degradation of cellular components. It is essential for the maintenance between synthesis, degradation and recycling of the cellular components [45]. For decades, autophagy has been known to be a cell-protective mechanism activated upon nutrient deprivation [23]. Other authors have also shown that L-arginine depletion may initiate autophagy as a protective early event, and inhibition of autophagy may enhance the therapeutic efficacy of L-arginine depletion treatment [24,25]. Starving cells may endure adverse conditions by reallocating nutrients through autophagy. BCA-M converts L-arginine into L-ornithine and urea, leading to L-arginine deprivation, which may, therefore, induce autophagy. Our results indicated that autophagy was significantly induced after 8 h of BCA-M treatment in HeLa and SiHa cells with positive and negative ASS expressions, respectively (Figure 8). The sign of autophagy induction after the treatment of BCA-M was similar to other reports with other types of arginine-depleting enzymes [18,46,47]. When autophagy is inhibited by CQ, the growth-inhibitory effect of BCA-M was enhanced synergistically (Table 2 and Figure 9). Thus, our results suggested that autophagy might serve as a cell protective mechanism and its induction preceded the growth inhibitory, apoptosis and cell cycle arrestment under BCA-M treatment in human cancer cells regardless of ASS expression.

## 4. Materials and Methods

### 4.1. Reagents and Materials

Materials not specified were obtained from Sigma-Aldrich (St. Louis, MO, USA). MTT (3-(4,5-dimethylthiazol-2-yl)-2,5-diphenyltetrazolium bromide) reagent, and all cell culture media, supplement and sera, were purchased from Invitrogen Life Technologies, Inc. (San Diego, CA, USA). Human cervical cancer cell lines HeLa, ME-180, C-33A and SiHa (ATCC numbers CCL-2, HTB-33, HTB-31, HTB-35, respectively) were obtained from American Type Culture Collection (ATCC; Manassas, VA, USA). Another human cervical cancer cell line CC-3 was obtained from the Chinese University of Hong Kong.

### 4.2. Preparation of Drugs

Recombinant *Bacillus caldovelox* arginase mutant (BCA-M) was prepared from *E. coli* harboring the expression vector for the his-tagged *Bacillus caldovelox* arginase and purified using nickel affinity column chromatography [10,48]. Recombinant L-arginine deiminase (ADI) were expressed and purified as described previously [49]. Activities of the BCA-M and ADI were determined using the Diacetyl Monoxime (DAMO) method [50,51], with 1 unit of the drugs defined as the amount needed to produce 1 μmol urea or L-citrulline, respectively, per min at 37 °C, pH 7.4. The specific activities of BCA-M and ADI were ~100 units per mg protein (U/mg) and ~25 U/mg, respectively.

### 4.3. Cell Culture

HeLa, ME-180, C-33A and SiHa cells were maintained in Dulbecco’s modified eagle’s medium (DMEM). Another human cervical cancer cell line CC-3 was cultured in a 1:1 (*v*/*v*) mixture of DMEM and Roswell park memorial institute 1640 medium (RPMI1640). All the culture media were supplemented with 10% fetal calf serum (FCS) and 100 units/mL penicillin/streptomycin (PS) and the cultures incubated at 37 °C in a humidified 5% CO_2_ incubator.

### 4.4. Cell Proliferation Assay

Five thousand cells from log-phase cultures were seeded in 100 μL per well of a 96-well plate (Iwaki, Toyko, Japan) separately and incubated at 37 °C in a humidified 5% CO_2_ incubator for 24 h before the culture medium was replaced with medium with or without a single or combination of drugs at various concentrations. The incubation was continued for 72 h and MTT assay was performed by adding 10 μL/well of 5 mg/mL MTT in PBS at 4 h before the end of incubation. Then, 100 μL/well of 10% SDS acidified with 0.01 N HCl was added and the plates were further incubated overnight. After complete dissolution, absorbance at 570 nm with reference to 655 nm was detected by a Benchmark microtiter plate reader (Bio-Rad Laboratories, Hercules, CA, USA). The absorbance of untreated cells was considered 100% and the drug doses needed to produce 50% growth inhibition (IC_50_) were calculated using GraphPad Prism 5 (GraphPad Software Inc., La Jolla, CA, USA) with non-linear regression sigmoidal dose–response curve model. Maximum percentage (Max. %) mean suppressions were determined within the tested dose ranges of the respective drugs. Combination index (C.I.) was calculated using the Chou-Talalay method [52] using IC_50_ values of drugs in the respective treatments with the formula

(1) $$C.I.=\frac{\mathrm{IC}50\;\mathrm{of}\;\mathrm{BCA}-M\;\mathrm{when}\;\mathrm{used}\;\mathrm{in}\;\mathrm{combination}}{\mathrm{IC}50\;\mathrm{of}\;\mathrm{BCA}-M\;\mathrm{when}\;\mathrm{used}\;\mathrm{alone}}\;\;\;\;+\frac{\mathrm{IC}50\;\mathrm{of}\;\mathrm{CQ}\;\mathrm{when}\;\mathrm{used}\;\mathrm{in}\;\mathrm{combination}}{\mathrm{IC}50\;\mathrm{of}\;\mathrm{CQ}\;\mathrm{when}\;\mathrm{used}\;\mathrm{alone}}$$

where C.I. value = 1.0 represents addition effect, while C.I. < 1 represents synergism and C.I. > 1 represents antagonism.

### 4.5. Reverse Transcription-Polymerase Chain Reaction (RT-PCR) Analysis

Total RNA was extracted using the RNeasy mini kit (Qiagen, Hilden, Germany) according to manufacturer’s instruction from cultured cells. For RT-PCR, cDNA was reverse-transcribed from 1 μg total RNA in a 20 μL reaction at 42 °C for 30 min using iScript™ cDNA synthesis kit (Bio-Rad Laboratories, Hercules, CA, USA) according to manufacturer’s instruction. Then, 1 μL of the cDNA was used as a template for PCR amplification using GoTaqGreen master mix (Promega, Madison, WI, USA) in a 20 μL reaction according to manufacturer’s instruction in a MyCycler thermocycler (Bio-Rad Laboratories). Primers used for OTC (5′-GATTTGGACACCCTGGCTAA-3′ and 5′-GGAGTAGCTGCCTGAAGGTG-3′, amplicon 221 bp) and glyceraldehyde-3-phosphate dehydrogenase gene (GAPDH) (5′-AGCCACATCGCTCAGACA-3′ and 5′-GCCCAATACGACCAAATCC-3′, amplicon 66 bp) were as described by [5]. The primers used for ASS (5′-GGGGTCCCTGTGAAGGTGACC-3′ and 5′-CGTTCATGCTCACCAGCTC-3′, amplicon 448 bp) and ASL (5′-CATCCCTTTGCGGACCAGGTA-3′ and 5′-CTCCTGATGACCCTCAAGGGA-3′, amplicon 219 bp) were as described by [3]. For Arg I (ARG1), primers 5′-GGCTGGTCTGCTTGAGAAAC-3′ and 5′-ATTGCCAAACTGTGGTCTCC-3′ were used (amplicon 216 bp). The amplification was performed for 27 cycles with annealing at 57 °C for 40 s and extension at 72 °C for 30 s. The reaction products were analyzed using 2% agarose gel electrophoresis and the band intensities determined by the ImageJ software (National Institutes of Health, Bethesda, MD, USA). The relative mRNA expression levels were estimated with normalization to GAPDH gene expression.

### 4.6. Western Blotting Analysis

Log-phase cultured cervical cancer cells without drug treatment were used for protein expression profiling and cells with or without treatment of BCA-M or the combination of BCA-M and CQ was used for LC3 protein detection. Total protein from these cells was extracted from cultured cells using lysis buffer (1% Igepal CA630, 150 mM NaCl, 50 mM Tris-Cl, pH 7.5) supplemented with 1× complete mini protease inhibitor cocktail (Roche Applied Science, Branford, CT, USA). For confirmation of OTC expression in OTC rescue study, transduced cells were lysed with Radio-immunoprecipitation assay (RIPA) lysis buffer (50 mM Tris-HCl, 150 mM NaCl, 1% Triton X-100, 1% sodium deoxycholate, 0.1% sodium dodecyl sulfate and 1 mM EDTA). To prepare rat liver for OTC positive control, a small piece of rat liver was resuspended in RIPA lysis buffer and passed through a 25 G needle several times and filtered. In each case, cell debris was removed by centrifugation at 12,000 rpm at 4 °C. The extracted protein was resolved using sodium dodecyl sulfate-polyacrylamide gel electrophoresis (SDS-PAGE) and electroblotted onto Polyvinylidene fluoride (PVDF) Immobilon-P membrane (Millipore, Bedford, MA, USA). The membranes were blocked with Tris-buffered saline (TTBS, 0.1% Tween-20, 100 mM Tris-Cl, pH 7.5, 0.9% NaCl) containing 5% blotting-grade blocker (Bio-Rad Laboratories) and the specific protein detected by hybridization with primary antibodies and visualized with appropriate horseradish peroxidase (HRP)-conjugated secondary antibodies using SuperSignal West Pico Chemiluminescent Substrate (Pierce Biotechnology, Rockford, IL, USA). For primary antibodies, mouse anti-ASS (1:5000, BD Biosciences, San Jose, CA, USA), rabbit anti-OTC (1:2500, Altas Antibodies, AB, USA), rabbit anti-LC3 (1:1000, MBL international, Woburn, MA, USA) and rabbit anti-GAPDH (1:5000, Cell Signaling Technology, Danvers, MA, USA) were used to detect the respective proteins. HRP-conjugated goat anti-mouse secondary antibody (1:2000, Santa Cruz Biotechnology, CA, USA) was used to detect anti-ASS. HRP-conjugated goat anti-rabbit secondary antibody (1:5000, Santa Cruz Biotechnology, CA, USA) was used to detect anti-LC3 antibody. Another HRP-conjugated goat anti-rabbit secondary antibody (Cell Signaling Technology, Danvers, MA, USA) was used to detect anti-OTC antibody (1:25,000) and anti-GAPDH antibody (1:80,000). Protein signal intensities were determined using the ImageJ software (National Institutes of Health).

### 4.7. Cell Cycle Analysis

Cervical cancer cells were treated with BCA-M at various concentrations for 48 or 72 h and then harvested by trypsinization, fixed in 60% ethanol at 4 °C, washed in PBS, filtered via 60 μm nylon mesh (Millipore, MA, USA), resuspended in PI/RNase staining buffer (BD Pharmingen, San Jose, CA, USA), and stained at 4 °C overnight. Finally, FACS analyses were performed on the stained cells using the FACSAria flow cytometer (BD Biosciences, San Jose, CA, USA). For each sample, data from 10,000 cells were collected and cell cycle phase distribution analyses were performed using ModFit LT 3.1 (Verity Software House, Topsham, ME, USA).

### 4.8. Quantitative Real-Time RT-PCR Analysis

Cervical cancer cells were seeded and treated as in cell cycle analysis. Total RNA was extracted, and cDNA was synthesized as described above. Then, quantitative real-time PCR was performed with the cDNA using ABI Power Syber Green PCR master mix (Applied Biosystems, Foster City, CA, USA) on an MyiQ and iQ5 real-time PCR Detection System (Bio-Rad Laboratories). Reactions for each gene of each sample were performed in triplicate and the gene expression levels were calculated using ΔΔCT method with normalization to GAPDH expression and relative to the normalized expression levels of the respective genes in the drug treatment control groups. Primers used for the cyclin A1 (CCNA1) (5′-AATGGGCAGTACAGGAGGAC-3′ and 5′-CCACAGTCAGGGAGTGCTTT-3′, amplicon 110 bp), cyclin B1 (CCNB1) (5′-CATGGTGCACTTTCCTCCTT-3′ and 5′-AGGTAATGTTGTAGAGTTGGTGTCC-3′, amplicon 102 bp), cyclin D1 (CCND1) (5′-GAAGATCGTCGCCACCTG-3′ and 5′-GACCTCCTCCTCGCACTTCT-3′, amplicon 61 bp) and cyclin E1 (CCNE1) (5′-GGCCAAAATCGACAGGAC-3′ and 5′-GGGTCTGCACAGACTGCAT-3′, amplicon 75 bp) were as described by [5]. For cyclin A2 (CCNA2) (5′-AAGTTTTCCTCTCAGCACTGAC-3′ and 5′-ACTACAGAATGAGACCCTGCATTTG-3′) were used (amplicon 82 bp) [53].

### 4.9. Apoptosis Induction Analysis

Cancer cells were treated with BCA-M at various concentrations and then harvested by trypsinization and washed in PBS. Detection of apoptosis was performed using the Annexin V-FITC Apoptosis Detection Kit (BD Biosciences, San Jose, CA, USA) according to manufacturer’s instruction. Briefly, the washed live cells were resuspended in binding buffer (10 mmol/L HEPES/NaOH (pH 7.4), 140 mmol/L NaCl, 2.5 mmol/L CaCl_2_), and stained with Annexin V-FITC and PI for over 15 min at room temperature in the dark. Finally, the cells were applied to FACS analysis using FACSAria flow cytometer (BD Biosciences, San Jose, CA, USA) within 1 h after staining.

### 4.10. Autophagy Detection

CYTO-ID^®^ Autophagy detection kit was used to monitor autophagy in live cancer cells by Leica TCS SPE confocal microscope following the manufacturer’s instructions (Enzo Life Sciences, Farmingdale, NY, USA).

### 4.11. Generation of Recombinant Adenoviruses for OTC Rescue

The recombinant adenoviruses used for OTC expression were generated using AdEasy Vector System (Stratagene, Santa Clara, CA, USA). Briefly, the OTC gene, amplified from human liver marathon-ready cDNA (Clontech Laboratories, Mountain View, CA, USA), was inserted between the restriction sites *NotI* and *EcoRV* of a transfer vector pShuttle-CMV using the forward primer 5′-AAGGAAAAAAGCGGCCGCATGCTGTTTAATCTGAGGATCCTGTTAAA-3′ and reverse primer 5′-CCGGATATCTCAAAATTTAGGCTTCTGGAGCTGAGGTGAGT-3′. The *EGFP* gene, isolated from vector pEGFP-C1 (Clontech Laboratories), was inserted into *KpnI* and *NotI* separately as control using the forward primer 5′-CCGGGTACCATGGTGAGCAAGGGCGAGGAGCTGTT-3′ and reverse primer 5′-AAGGAAAAAAGCGGCCGCTCACTTGTACAGCTCGTCCATGCCGAGAGT-3′. The resulting plasmid was then linearized with *PmeI* and cotransformed with plasmid pADEasy-1 into *E. coli* strain BJ5183 for homologous recombination. After screening for successful recombination and amplification using XL10 gold ultracompetent cells for greater DNA yield, the recombinant adenovirus construct was digested with *PacI* and transfected into AD293 cells by FugeneHD (Promega, Madison, WI, USA) for viral particle production. The effect of OTC expression was tested with cell proliferation assay in cancer cells transduced at 1:50 MOI with the recombinant adenovirus 24 h before addition of drug. The expression of OTC in transduced cells was also confirmed by Western blotting.

### 4.12. Statistical Analysis

Statistical analysis was performed with one-way ANOVA with post hoc Dunnett’s test and *p* values < 0.05 were considered statistically significant.

## 5. Conclusions

BCA-M significantly inhibited the growth of human cervical cancer cells in vitro regardless of the expression of ASS, while the ASS expression is strongly associated with the susceptibility of cancer cells towards ADI. BCA-M, therefore, offers the advantage of a broader spectrum of susceptible cancer cells. Drug susceptibilities correlate well with the expressions of major urea cycle genes and completeness of L-arginine regeneration pathways. Mechanistic studies showed that autophagy served as a protective mechanism and the growth inhibitory effects of BCA-M could be enhanced synergistically by its combination with the autophagy inhibitor, CQ, regardless of ASS expression. On the other hand, the anticancer mechanisms of BCA-M involved efficient induction of apoptosis and cell cycle arrest at S and/or G_2_/M phases. Thus, BCA-M resulted in multiple suppression mechanisms towards all tested cancers, meaning that BCA-M may be a promising candidate for the treatment of cervical cancers.

## Figures and Tables

**Table 1:** IC_50_ values, maximum percentage mean suppression and relative gene expression profile of human cervical cancer cells.

Cell Line	BCA-M	ADI	Relative Gene Expression ^b^				
	IC_50_ Value ^a^, U/mL (Max. %)	IC_50_ Value ^a^, Milli-Units/mL (Max. %)	GAPDH	Arg I	OTC	ASL	ASS
C-33A	0.19 ± 0.06 (85.0%)	1.47 ± 0.7 (71.0%)	1.00	UD	UD	0.22	UD
SiHa	0.31 ± 0.12 (78.1%)	2.99 ± 2.12 (73.0%)	1.00	UD	UD	0.50	UD
HeLa	0.53 ± 0.13 (70.9%)	N/A (32.6%)	1.00	UD	UD	0.35	0.19
CC3	1.36 ± 0.89 (58.9%)	N/A (0.4%)	1.00	UD	UD	0.18	0.66
ME-180	1.42 ± 0.33 (65.4%)	N/A (8.1%)	1.00	UD	UD	0.80	2.31

^a^ IC_50_ values expressed as mean ± S.D. were determined from MTT-based cell proliferation assays where N/A represent that the cell line was resistant to the drug treatment and no IC_50_ value can be calculated. Maximum percentage mean suppressions (Max. %) were determined within the tested dose ranges of the respective drugs and shown within brackets below the IC_50_ values. ^b^ Relative gene expression profile for the mRNA of the respective genes was determined using semi-quantitative RT-PCR and analyzed using agarose gel electrophoresis with the levels normalized by that of the internal control glyceraldehyde-3-phosphate dehydrogenase (GAPDH). UD represent that the relative gene expression was undetermined due to undetectable expression.

**Table 2:** IC_50_ values and combination index (C.I.) for the treatment of BCA-M and chloroquine.

Cell	Treatment	Drug	IC_50_ Value ^a^	C.I.
SiHa	BCA-M alone	BCA-M	0.31 ± 0.12 U/mL	
	CQ alone	CQ	25.7 ± 4.7 μM	
	BCA-M + CQ	BCA-M	0.17 ± 0.05 U/mL	0.78 ± 0.06
		CQ	3.37 ± 0.95 μM	
HeLa	BCA-M alone	BCA-M	0.53 ± 0.13 U/mL	
	CQ alone	CQ	27.1 ± 6.2 μM	
	BCA-M + CQ	BCA-M	0.30 ± 0.09 U/mL	0.70 ± 0.21
		CQ	1.49 ± 0.46 μM	

^a^ IC_50_ values expressed as mean ± S.D. were determined from MTT-based cell proliferation assays. Combination indexes (C.I.) were calculated using the Chou-Talalay method from the IC_50_ values of drugs in the respective treatments. C.I. < 1 represents synergistic effect.

## Supplementary Materials

The following are available online at https://www.mdpi.com/1422-0067/21/20/7445/s1, Figure S1: Representative results showing the effect of BCA-M on cell cycle phase distribution of HeLa cells determined using flow cytometric analysis with propidium iodide (PI) staining and RNase digestion. Figure S2: Representative results showing the effect of BCA-M on cell cycle phase distribution of ME-180 cells determined using flow cytometric analysis with propidium iodide (PI) staining and RNase digestion; Figure S3: Representative results showing the effect of BCA-M on cell cycle phase distribution of SiHa cells determined using flow cytometric analysis with propidium iodide (PI) staining and RNase digestion; Figure S4: Representative results showing the effect of BCA-M on apoptosis induction in C-33A cells determined using flow cytometric analysis with Annexin V-FITC and propidium iodide (PI) staining; Figure S5. Representative results showing the effect of BCA-M on apoptosis induction in SiHa cells determined using flow cytometric analysis with Annexin V-FITC and propidium iodide (PI) staining; Figure S6. Representative results showing the effect of BCA-M on apoptosis induction in HeLa cells determined using flow cytometric analysis with Annexin V-FITC and propidium iodide (PI) staining; Figure S7. Representative results showing the effect of BCA-M on apoptosis induction in ME-180 cells determined using flow cytometric analysis with Annexin V-FITC and propidium iodide (PI) staining; Figure S8. HeLa and SiHa cells were treated with 0.4–1.6 U/ml of BCA-M for 1 h; Figure S9. Growth inhibition of CQ on HeLa and SiHa human cervical cancer cells.
