# Supplementary material for: Recombinant Bacillus caldovelox Arginase Mutant (BCA-M) Induces Apoptosis, Autophagy, Cell Cycle Arrest and Growth Inhibition in Human Cervical Cancer Cells
Source: Int J Mol Sci. 2020 Oct 9;21(20):7445. doi: 10.3390/ijms21207445 (PMC7589785; doi:10.3390/ijms21207445)
Supplement: Supplementary file 1 [file ijms-21-07445-s001.pdf]

International Journal of *Molecular Sciences*

MDPI

Supporting Information

Recombinant *Bacillus Caldovelox* Arginase Mutant (BCA-M) Induces Apoptosis, Autophagy, Cell Cycle Arrest and Growth Inhibition in Human Cervical Cancer Cells

Sai-Fung Chung †, Chi-Fai Kim †, Ho-Yin Chow, Hiu-Chi Chong, Suet-Ying Tam, Yun-Chung Leung * and Wai-Hung Lo *

Department of Applied Biology and Chemical Technology, Lo Ka Chung Research Centre for Natural Anti-Cancer Drug Development and State Key Laboratory of Chemical Biology and Drug Discovery, The Hong Kong Polytechnic University, Hung Hom, Kowloon, Hong Kong, China; 16900402r@connect.polyu.hk (S.-F.C.); stephen.kim@polyu.edu.hk (C.-F.K.); hoyin.chow@polyu.edu.hk (H.-Y.C.) steve.h.c.chong@gmail.com (H.-C.C.);sabrinasy.tam@connect.polyu.hk (S.-Y.T.)

* Correspondence: thomas.yun-chung.leung@polyu.edu.hk (Y.-C.L.); thomas.wai-hung.lo@polyu.edu.hk (W.-H.L.); Tel.: +852-3400-8661 (Y.-C.L.)

† These authors contributed equally to this work.

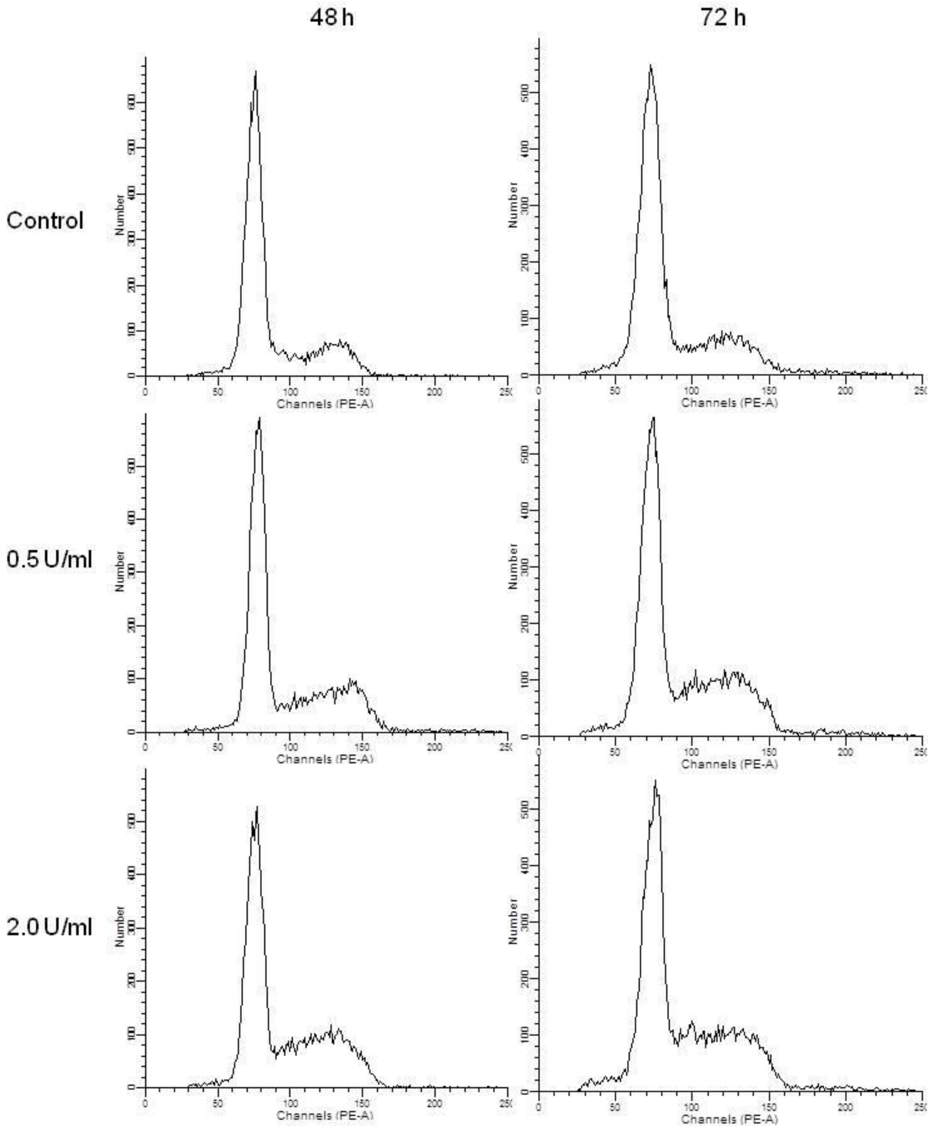

Figure S1. Representative results showing the effect of BCA-M on cell cycle phase distribution of HeLa cells determined using flow cytometric analysis with propidium iodide (PI) staining and RNase digestion. The results of no. of events plot against PI signal intensity were plot as histograms.

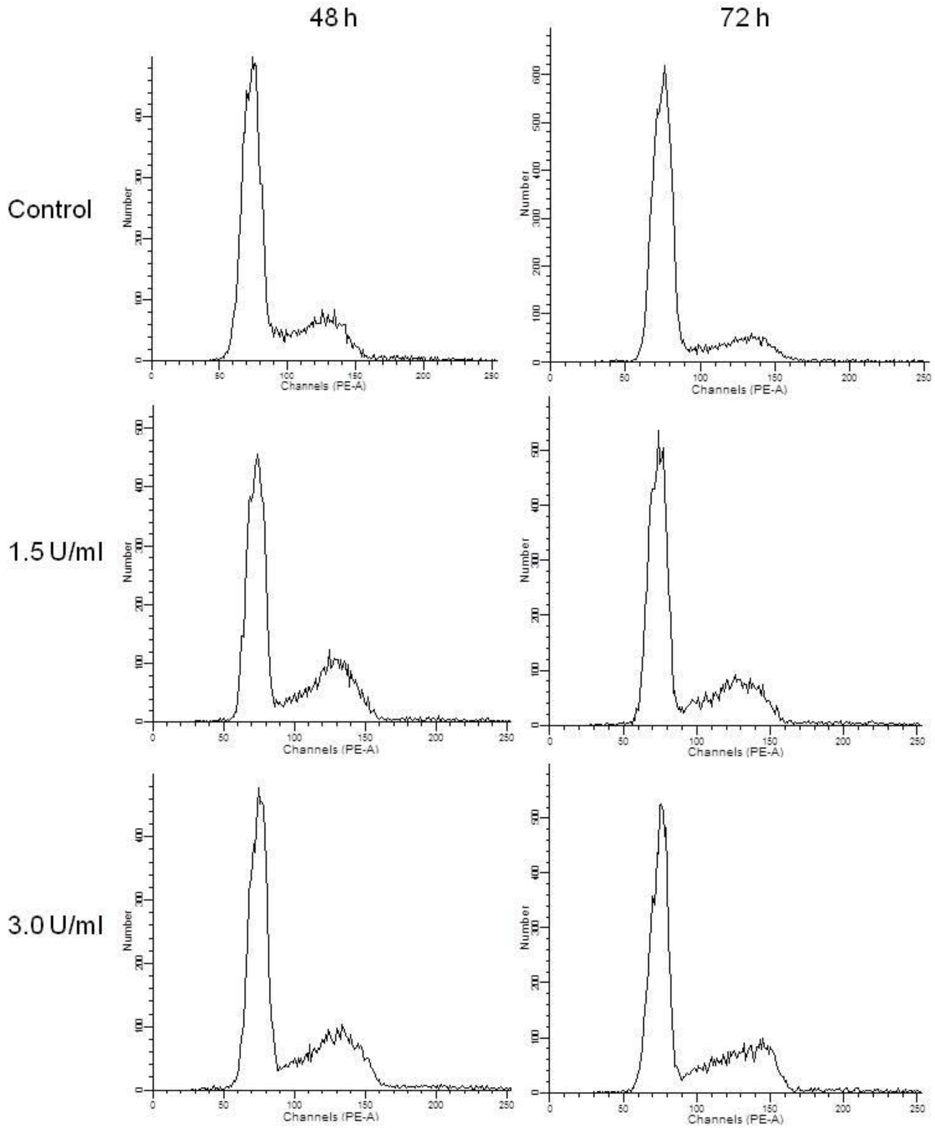

Figure S2. Representative results showing the effect of BCA-M on cell cycle phase distribution of ME-180 cells determined using flow cytometric analysis with propidium iodide (PI) staining and RNase digestion. The results of no. of events plot against PI signal intensity were plot as histograms.

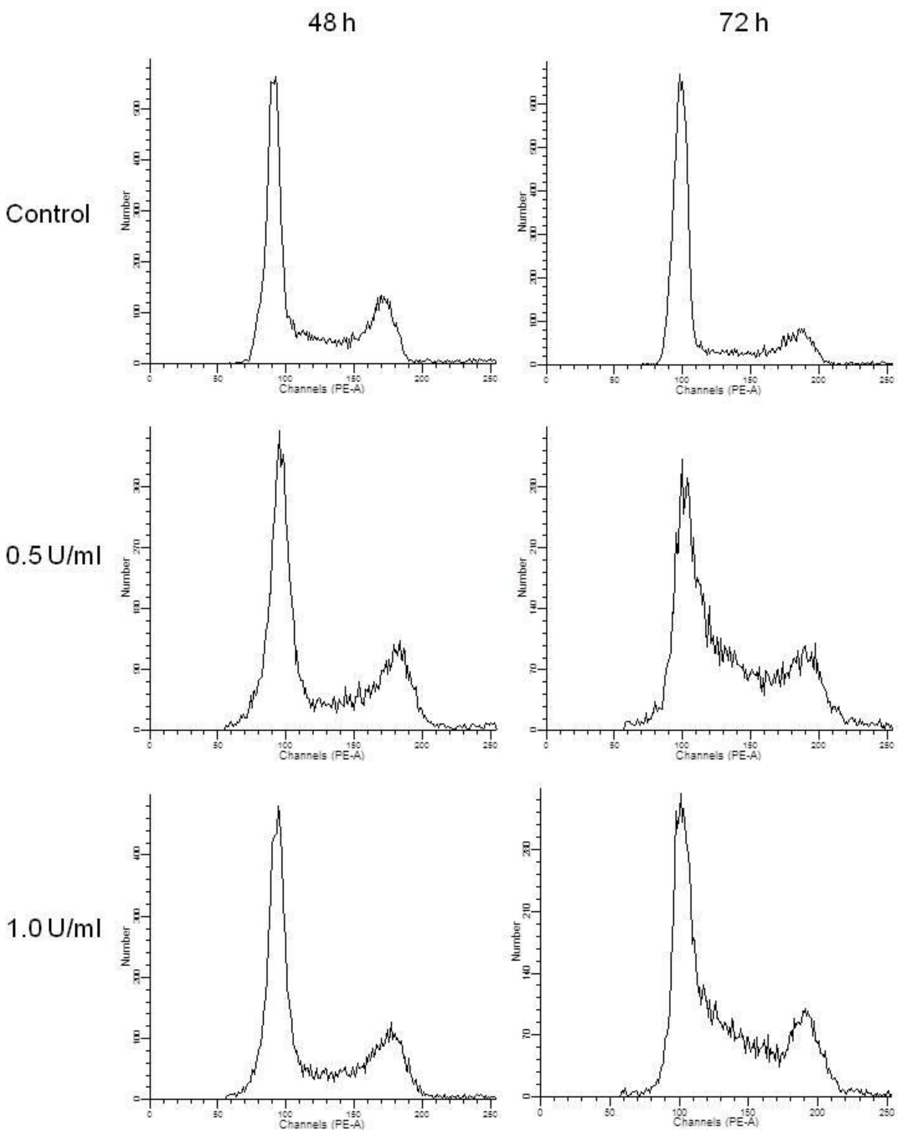

Figure S3. Representative results showing the effect of BCA-M on cell cycle phase distribution of SiHa cells determined using flow cytometric analysis with propidium iodide (PI) staining and RNase digestion. The results of no. of events plot against PI signal intensity were plot as histograms.

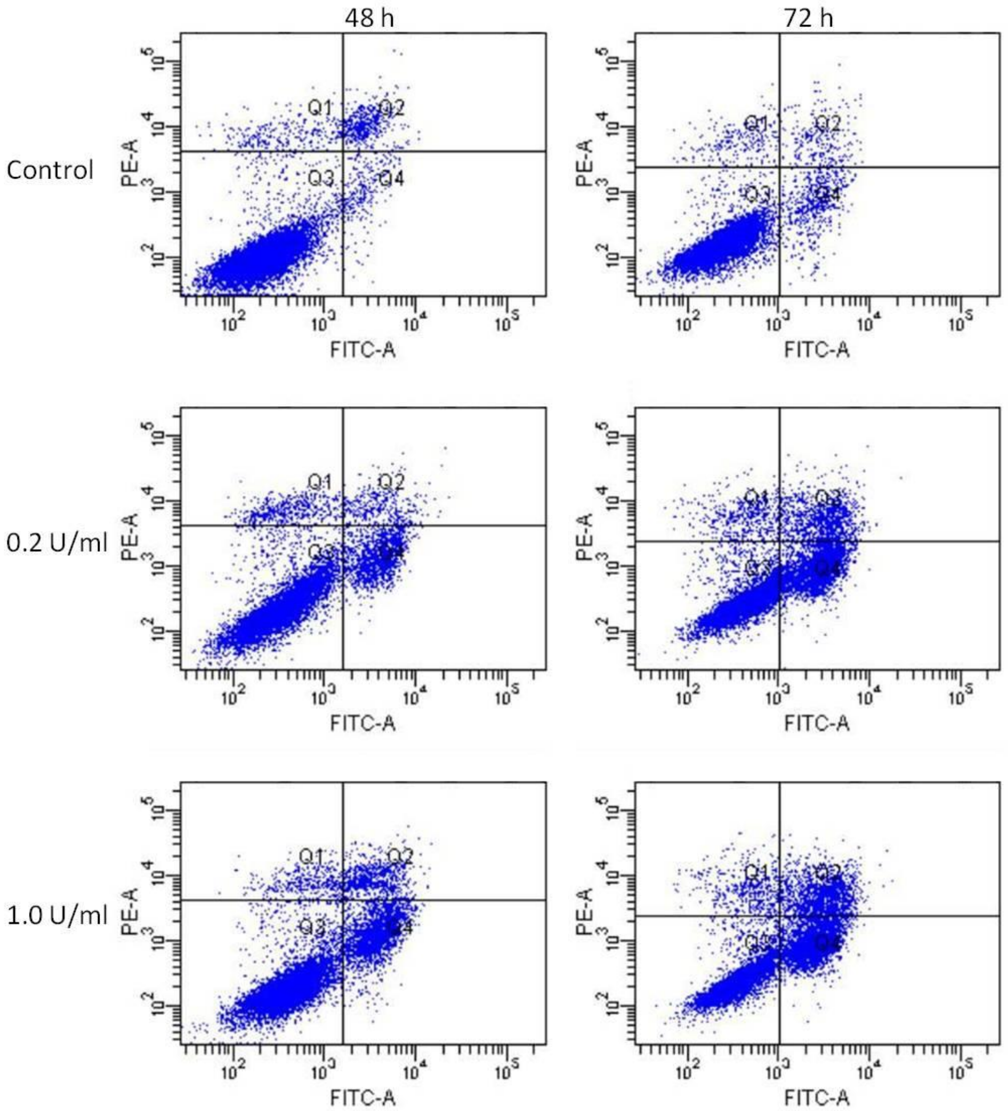

Figure S4. Representative results showing the effect of BCA-M on apoptosis induction in C-33A cells determined using flow cytometric analysis with Annexin V-FITC and propidium iodide (PI) staining. The results were expressed in density plots with signal intensity of PI against FITC.

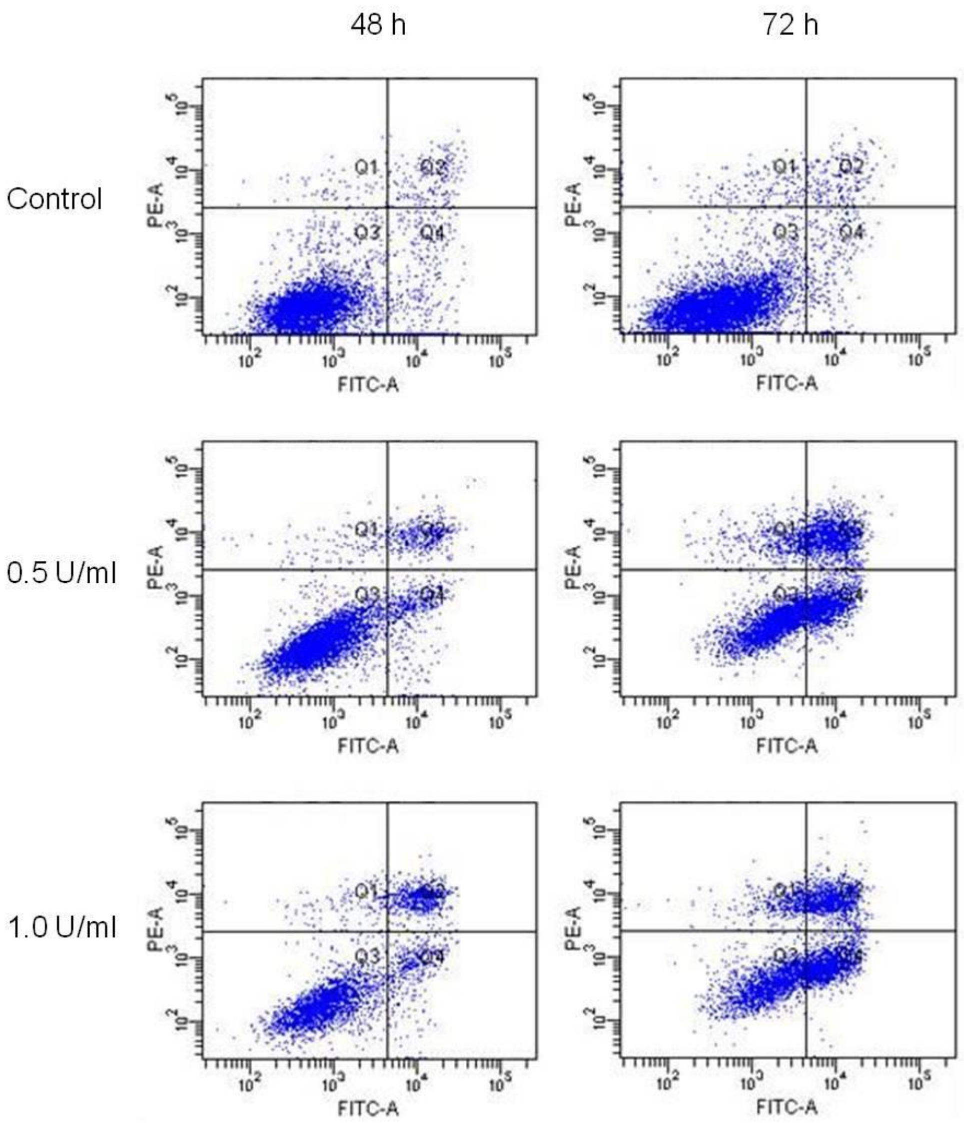

Figure S5. Representative results showing the effect of BCA-M on apoptosis induction in SiHa cells determined using flow cytometric analysis with Annexin V-FITC and propidium iodide (PI) staining. The results were expressed in density plots with signal intensity of PI against FITC.

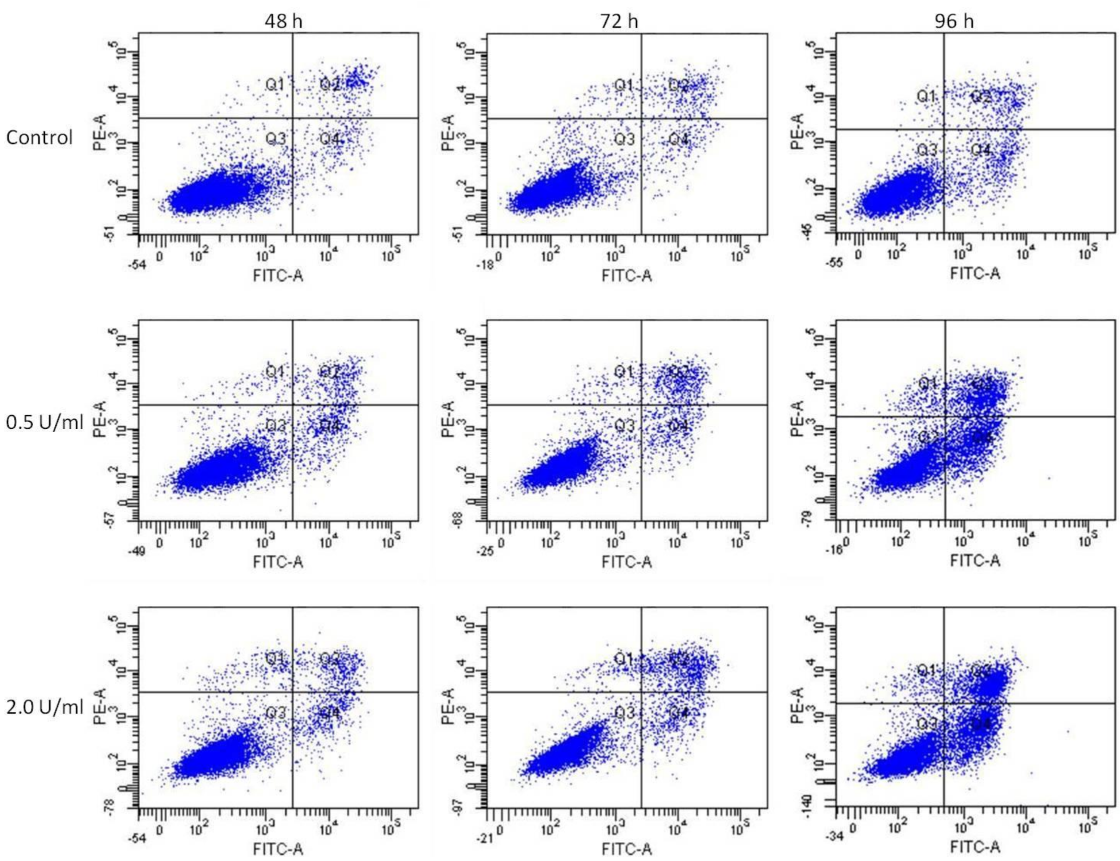

Figure S6. Representative results showing the effect of BCA-M on apoptosis induction in HeLa cells determined using flow cytometric analysis with Annexin V-FITC and propidium iodide (PI) staining. The results were expressed in density plots with signal intensity of PI against FITC.

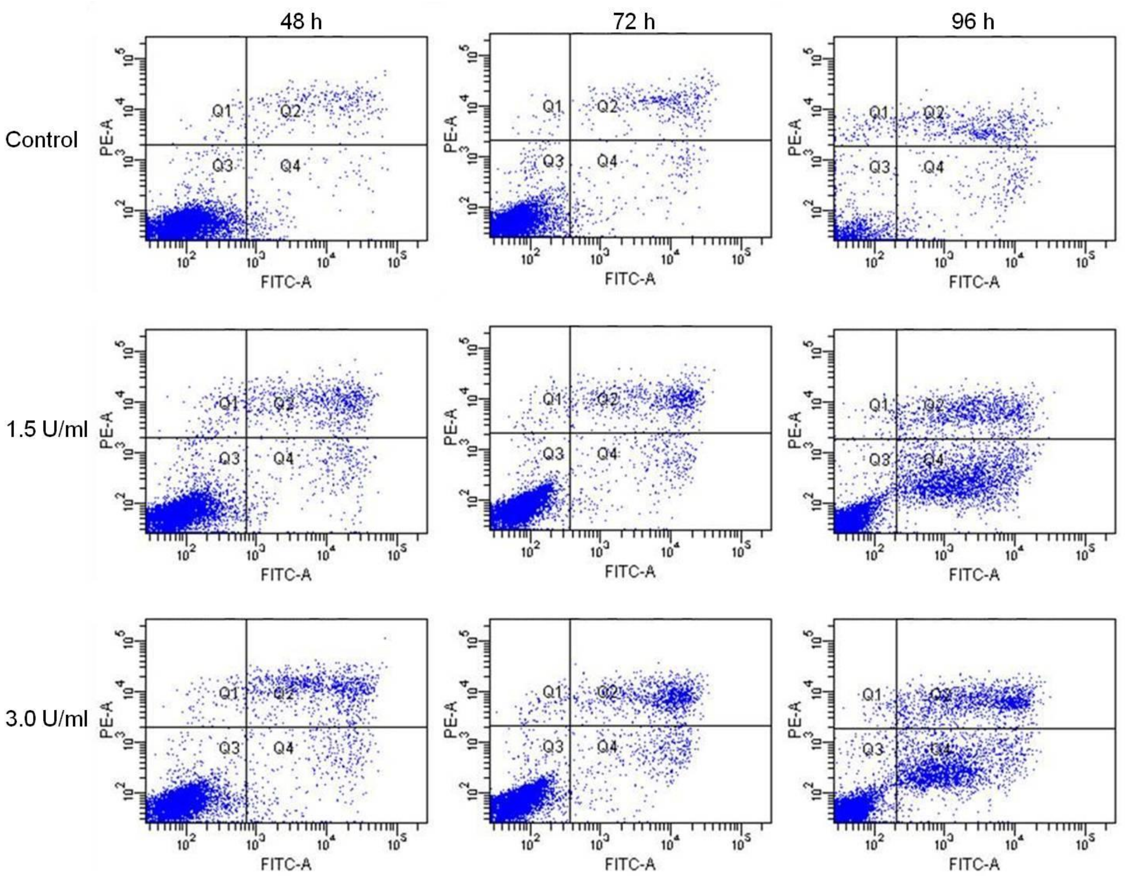

Figure S7. Representative results showing the effect of BCA-M on apoptosis induction in ME-180 cells determined using flow cytometric analysis with Annexin V-FITC and propidium iodide (PI) staining. The results were expressed in density plots with signal intensity of PI against FITC.

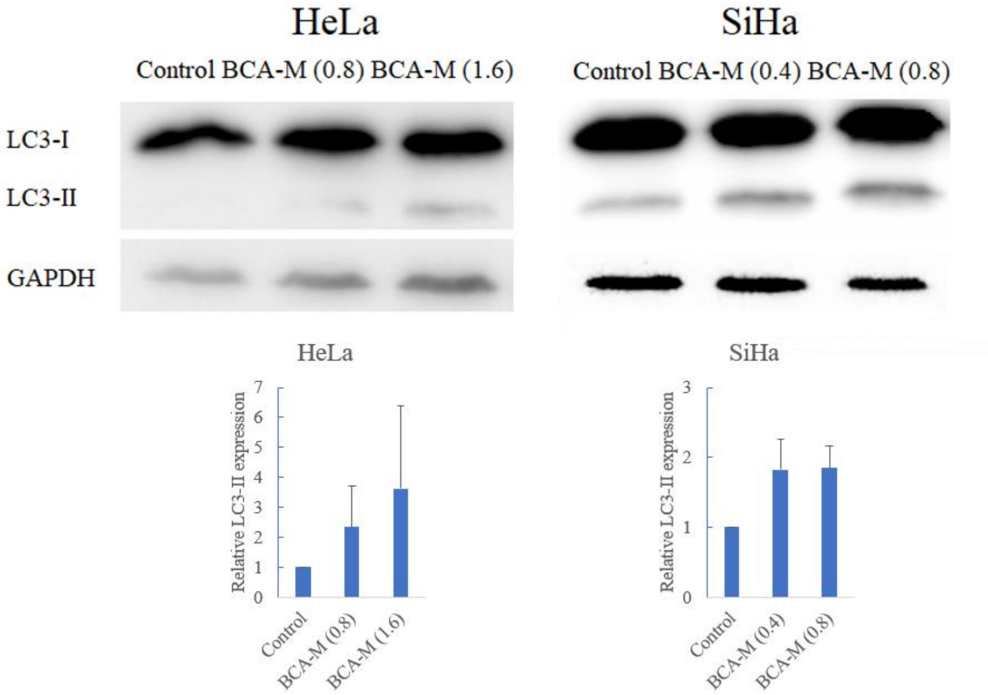

Figure S8. HeLa and SiHa cells were treated with 0.4–1.6 U/ml of BCA-M for 1 hour. The data are presented as means ± S.D. of three independent experiments.

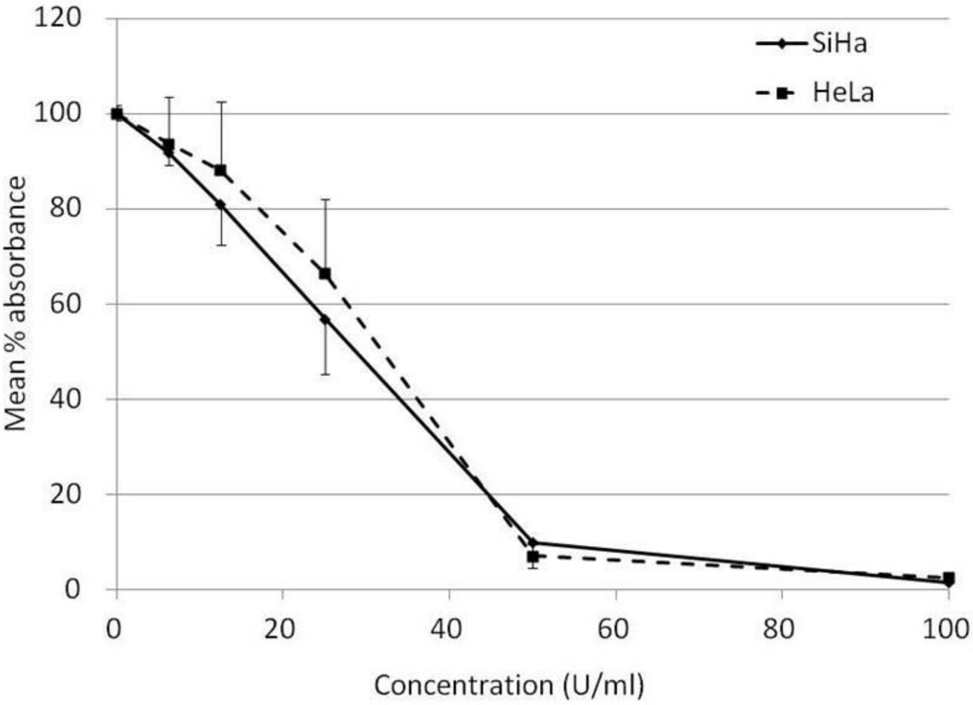

Figure S9. Growth inhibition of CQ on HeLa and SiHa human cervical cancer cells. The results of cell proliferation assay are represented by the mean % absorbance and expressed as percentage mean with error bar showing S.D. on a single side.
